# The phospholipase PNPLA7 functions as a lysophosphatidylcholine hydrolase and interacts with lipid droplets through its catalytic domain

**DOI:** 10.1074/jbc.M117.792978

**Published:** 2017-09-07

**Authors:** Christoph Heier, Benedikt Kien, Feifei Huang, Thomas O. Eichmann, Hao Xie, Rudolf Zechner, Ping-An Chang

**Affiliations:** From the ‡ Institute of Molecular Biosciences, University of Graz, 8010 Graz, Austria,; ¶ BioTechMed-Graz, 8010 Graz, Austria, and; § Key Laboratory of Molecular Biology, School of Bio-information, Chongqing University of Posts and Telecommunications, Chongqing 400065, China

**Keywords:** endoplasmic reticulum (ER), lipid droplet, lysophospholipid, phospholipase, phospholipid metabolism, protein domain

## Abstract

Mammalian patatin-like phospholipase domain–containing proteins (PNPLAs) are lipid-metabolizing enzymes with essential roles in energy metabolism, skin barrier development, and brain function. A detailed annotation of enzymatic activities and structure–function relationships remains an important prerequisite to understand PNPLA functions in (patho-)physiology, for example, in disorders such as neutral lipid storage disease, non-alcoholic fatty liver disease, and neurodegenerative syndromes. In this study, we characterized the structural features controlling the subcellular localization and enzymatic activity of PNPLA7, a poorly annotated phospholipase linked to insulin signaling and energy metabolism. We show that PNPLA7 is an endoplasmic reticulum (ER) transmembrane protein that specifically promotes hydrolysis of lysophosphatidylcholine in mammalian cells. We found that transmembrane and regulatory domains in the PNPLA7 N-terminal region cooperate to regulate ER targeting but are dispensable for substrate hydrolysis. Enzymatic activity is instead mediated by the C-terminal domain, which maintains full catalytic competence even in the absence of N-terminal regions. Upon elevated fatty acid flux, the catalytic domain targets cellular lipid droplets and promotes interactions of PNPLA7 with these organelles in response to increased cAMP levels. We conclude that PNPLA7 acts as an ER-anchored lysophosphatidylcholine hydrolase that is composed of specific functional domains mediating catalytic activity, subcellular positioning, and interactions with cellular organelles. Our study provides critical structural insights into an evolutionarily conserved class of phospholipid-metabolizing enzymes.

## Introduction

Mammalian patatin-like phospholipase domain–containing proteins (PNPLAs)^2^ constitute a family of lipid-metabolizing enzymes with critical roles in energy metabolism, skin barrier development, and brain function (1–4). The patatin-like phospholipase domain (Pfam01734) is named after a homologous domain in the potato protein patatin, which adopts a three-layer α/β/α architecture and harbors an enzymatic active site composed of a Ser-Asp catalytic dyad (1, 5). In accordance with a primarily enzymatic function of the patatin-like phospholipase domain, mammalian PNPLAs have been assigned hydrolase, transacylase, or acyltransferase activities with diverse lipid substrates such as phospholipids, acylglycerols, and retinoids (6–9). These reactions have been proven essential for the turnover of cellular membranes, mobilization of storage lipids, and generation of signaling molecules (10–12). The physiological relevance of PNPLAs is illustrated by a diverse spectrum of inherited disorders that have been associated with mutations in human *PNPLA* genes including neutral lipid storage disease, non-alcoholic fatty liver disease, ichthyosis, hereditary spastic paraplegia, and other neurodegenerative syndromes (2, 3, 13, 14). Of note, defects in enzymatic function and subcellular localization have been identified as common molecular mechanisms in the onset and progression of PNPLA-related disorders (4, 15, 16).

PNPLA6 and PNPLA7 constitute a subgroup within the PNPLA family that has been remarkably conserved during evolution with orthologous proteins in yeast, nematodes, and flies (17). PNPLA6 acts as a (lyso)phospholipase and is involved in the degradation of membrane lipids such as phosphatidylcholine (PC) and lysophosphatidylcholine (LPC) (6, 11). Human PNPLA6 is a primary substrate of organophosphates (OPs) that cause a neurotoxic syndrome termed OP-induced delayed neuropathy. Inhibition of PNPLA6 activity and the resultant disruption of neuronal phospholipid homeostasis initiate OP-induced delayed neuropathy. Accordingly, PNPLA6 has been traditionally referred to as neuropathy target esterase (18, 19). Recently, mutations in the human *PNPLA6* gene have been linked to a complex spectrum of neuroendocrine disorders including ataxia, spastic paraplegia, chorioretinopathy, and hypopituitarism (4, 14, 20, 21). Similar neurodegenerative phenotypes have been observed in PNPLA6 mutant animals from diverse phyla, indicating an evolutionarily conserved role of PNPLA6-mediated phospholipid catabolism in brain function (22–24).

In contrast to PNPLA6, little is known about the molecular and physiological function(s) of the closely related PNPLA7 (also termed neuropathy target esterase-related esterase). Both proteins share a highly conserved domain architecture that is assembled of the enzymatic patatin-like phospholipase domain and extensive “non-enzymatic” segments of poorly defined function including three putative cyclic nucleotide monophosphate (cNMP)-binding sites (1, 25). Initial *in vitro* studies identified PNPLA7 as a lysophospholipase whose transcript expression is highly responsive to feeding/fasting transitions and insulin concentrations (25, 26). In mammalian cells, PNPLA7 localizes to the ER and lipid droplets (LDs), which are cellular lipid storage organelles with pivotal functions in energy metabolism and lipid trafficking (25, 27). Although these observations closely link PNPLA7 to lipid and energy metabolism, it is presently unknown how fluctuations in PNPLA7 expression or subcellular distribution affect lipid homeostasis of cells or tissues (25).

In this study, we further characterized the enzymatic function of PNPLA7 in cellular lipid metabolism and established detailed structure–function relationships among domain architecture, subcellular positioning, and enzymatic activity of the protein. We confirm that PNPLA7 acts as a potent intracellular lysophospholipase and identify LPC as a major substrate of PNPLA7 in living cells. Moreover, we demonstrate that PNPLA7 is composed of specific functional parts mediating ER targeting, interactions with LDs, and substrate hydrolysis. Our study provides novel structural insights into an evolutionarily conserved class of phospholipid-metabolizing enzymes.

## Results

### PNPLA7 expression affects lysophospholipid metabolism in mammalian cells

**Figure 1.**
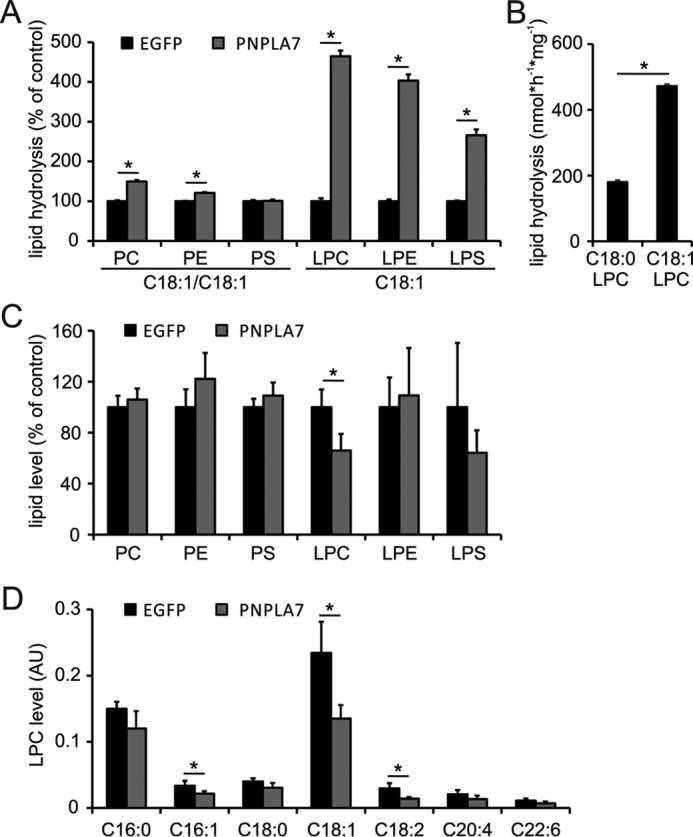
**Effects of PNPLA7 overexpression on cellular lipid metabolism.** *A* and *B*, lipid hydrolase activities of PNPLA7-EGFP. PNSs of COS-7 cells stably expressing PNPLA7-EGFP or EGFP were incubated with glycerophospholipid substrates containing C18:1 or C18:0 FAs as indicated, and the release of FAs was determined. *C* and *D*, lipid levels of COS-7 cells stably expressing PNPLA7-EGFP or EGFP. Total lipids were extracted, and the cellular levels of the indicated lipids were analyzed by LC/MS. Data are representative of two independent experiments and are expressed as means (*n* = 3). *Error bars* represent S.D. Statistical significance was determined using Student's unpaired *t* test. *, *p* < 0.05. *AU*, arbitrary units.

**Figure 2.**
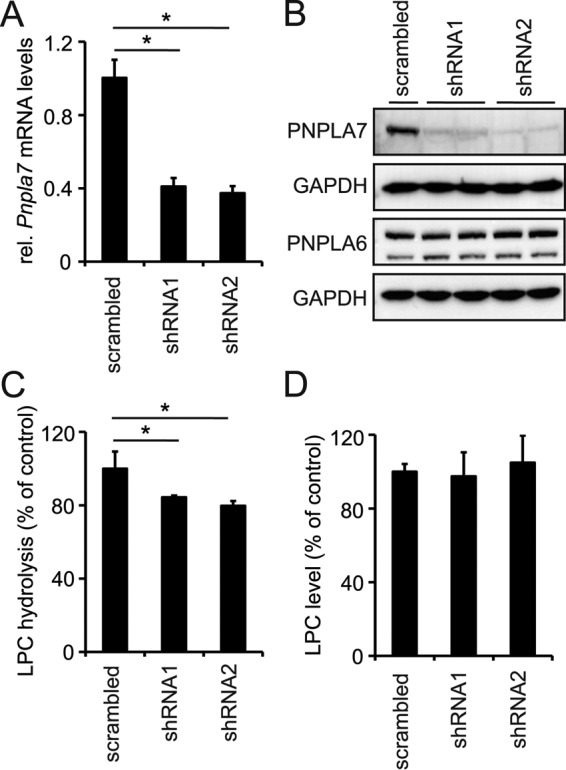
**Effects of PNPLA7 knockdown on cellular lipid metabolism.** *A*, *Pnpla7* mRNA levels in AML12 cells stably expressing individual shRNAs targeting *Pnpla7* (shRNA1 and shRNA2). Cells stably expressing scrambled shRNA served as control. Relative (*rel*.) mRNA levels were quantified by RT-qPCR and are normalized to *36B4. B*, protein levels of PNPLA7, PNPLA6, and GAPDH in AML12 cells expressing shRNA1, shRNA2, or scrambled shRNA. PNSs of AML12 cells were subjected to immunoblotting using antibodies against PNPLA7 (R domain), PNPLA6, or GAPDH. *C*, LPC hydrolase activities of AML12 cells expressing shRNA1, shRNA2, or scrambled shRNA. PNSs of AML12 cells were incubated with LPC, and the release of FAs was quantified. *D*, LPC levels of AML12 cells stably expressing shRNA1, shRNA2, or scrambled shRNA. Total lipids were extracted and analyzed by LC/MS. Data are representative of two independent experiments. Data are expressed as means, and *error bars* represent S.D. (*n* = 3–4). Statistical significance was determined using Student's unpaired *t* test. *, *p* < 0.05.

To assess possible function(s) of PNPLA7 in cellular lipid metabolism, we first created cell lines stably expressing PNPLA7-EGFP or EGFP, respectively, and subjected them to enzyme activity assays and lipid analysis. Consistent with previous studies, cell homogenates expressing PNPLA7-EGFP exhibited increased hydrolytic activity toward several lysophospholipid species as compared with EGFP-expressing control homogenates including C18:1 LPC (4.6-fold), C18:1 lysophosphatidylethanolamine (LPE; 4.0-fold), and C18:1 lysophosphatidylserine (LPS; 2.7-fold) (25). In addition, PNPLA7-EGFP-expressing homogenates showed a low but significant increase in the hydrolytic activity toward C18:1/C18:1 PC (1.5-fold) and C18:1/C18:1 phosphatidylethanolamine (PE; 1.2-fold), whereas the activity toward C18:1/C18:1 phosphatidylserine (PS) was not different from EGFP-expressing controls (Fig. 1*A*). The lipid hydrolase activity of PNPLA7-EGFP was 2.6-fold higher toward C18:1 LPC as compared with C18:0 LPC, suggesting a preference of the enzyme for unsaturated compared with saturated species (Fig. 1*B*). Lipid analysis revealed that stable expression of PNPLA7-EGFP significantly reduced cellular LPC levels (−34%) as compared with EGFP-expressing control cells but did not affect total cellular levels of LPE, LPS, PC, PE, or PS (Fig. 1*C*). The decrease in LPC levels observed in PNPLA7-EGFP-expressing cells was mainly due to a reduction in unsaturated LPC species containing C16:1 (−35%), C18:1 (−43%), and C18:2 (−52%) fatty acids (FAs) (Fig. 1*D*). Although a trend toward decreased cellular levels was also observed for other LPC species, the difference was not significant when compared with controls (Fig. 1*D*). To further investigate the function of PNPLA7 in cellular LPC metabolism, we depleted endogenous PNPLA7 in murine AML12 cells by RNAi and assessed cellular LPC hydrolase activities and LPC levels. Stable expression of two independent shRNA constructs targeting PNPLA7 (shRNA1 and shRNA2) decreased cellular *Pnpla7* mRNA concentrations by 59 and 63%, respectively, as compared with cells expressing scrambled shRNA (Fig. 2*A*). This was accompanied by decreased PNPLA7 protein levels in cells expressing either shRNA1 or shRNA2, whereas expression of the closely related PNPLA6 was unchanged (Fig. 2*B*). The reduction in PNPLA7 expression was associated with a 16–20% decrease in cellular LPC hydrolase activity (depending on the shRNA used; Fig. 2*C*) but did not affect total cellular LPC levels (Fig. 2*D*). Taken together, these data provide evidence that PNPLA7 acts as an intracellular LPC-preferring lysophospholipase that contributes to cellular LPC hydrolase activity in mammalian cells.

### PNPLA7 is an integral membrane protein with a luminal N and a cytosolic C terminus

**Figure 3.**
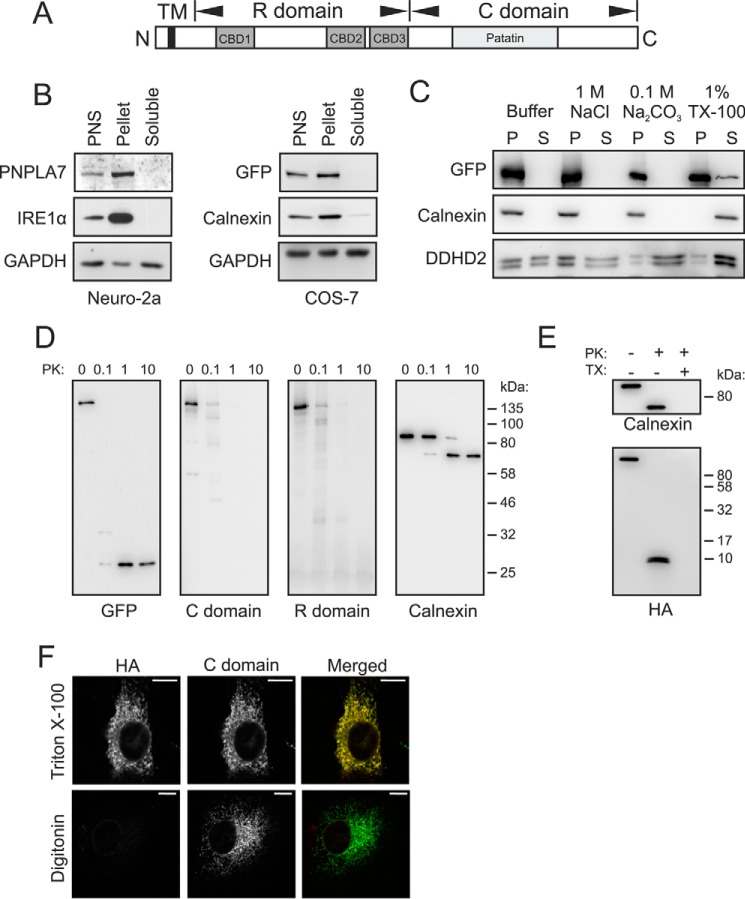
**Domain architecture, subcellular localization, and membrane topology of PNPLA7.** *A*, predicted domains of PNPLA7. The N-terminal region of PNPLA7 comprises a putative transmembrane domain (*black*) and three putative CBDs, which are designated as the R domain. The C domain contains a patatin domain implicated in the enzymatic function. *B*, distribution of endogenous PNPLA7 and ectopically expressed PNPLA7-EGFP in subcellular fractions. PNSs of Neuro-2a cells and COS-7 cells expressing PNPLA7-EGFP were fractionated into cytosol and membranes, and the distribution of proteins was assessed by immunoblotting using antibodies against the R domain of PNPLA7, GFP, IRE1α, Calnexin, and GAPDH. *C*, membrane association of PNPLA7-EGFP. Membranes of COS-7 cells expressing PNPLA7-EGFP were treated with the indicated agents, and soluble (*S*) and pellet (*P*) fractions were obtained by ultracentrifugation and analyzed by immunoblotting using antibodies against GFP, Calnexin, and DDHD2. *D* and *E*, sensitivity of PNPLA7 epitopes to protease treatment. Membrane fractions of COS-7 cells expressing PNPLA7-EGFP (*D*) or HA-PNPLA7 (*E*) were incubated in the presence of 0–10 μg/ml proteinase K (*D*) or 10 μg/ml proteinase K (*PK*) and 1% Triton X-100 (*TX*) as indicated (*E*) and subjected to immunoblotting using antibodies against GFP, the R domain of PNPLA7, the C domain of PNPLA7, HA, or Calnexin as indicated. *F*, immunostaining of PNPLA7 epitopes in COS-7 cells. COS-7 cells expressing HA-PNPLA7 were fixed, permeabilized with Triton X-100 or digitonin as indicated, and costained with antibodies directed toward the N-terminal HA tag and the C domain of PNPLA7. *Scale bars*, 10 μm.

To link the lipid-metabolizing function of PNPLA7 to a specific cellular site, we next assessed its subcellular localization and the topological orientation of its domains. The arrangement of possible functional domains within the PNPLA7 polypeptide is shown in Fig. 3*A*. The N-terminal half of the protein includes a single predicted transmembrane (TM) domain close to the N terminus and a putative “regulatory” (R) domain, which harbors three predicted cNMP-binding domains (CBDs). The C-terminal half (C domain) includes the patatin-like phospholipase domain predicted to mediate enzymatic activity (Fig. 3*A*). Subcellular fractionation of Neuro-2a postnuclear supernatants (PNSs) and subsequent immunoblotting showed that endogenous PNPLA7 was enriched in the membrane fraction but undetectable in the cytosol (Fig. 3*B*). Likewise, a PNPLA7-EGFP fusion protein transiently expressed in COS-7 cells was exclusively recovered in the membrane fraction (Fig. 3*B*), indicating a similar subcellular distribution of endogenous PNPLA7 and ectopically expressed PNPLA7-EGFP. Membrane-associated PNPLA7-EGFP was partially solubilized with Triton X-100 but not with NaCl or Na_2_CO_3_, similar to the integral membrane protein Calnexin but unlike the peripheral membrane protein DDHD2 (28, 29) (Fig. 3*C*). Next, we used proteinase K protection assays to assess the topological orientation of PNPLA7 within cellular membranes. Incubation of isolated microsomes with increasing concentrations of proteinase K provoked a dose-dependent depletion of the signal corresponding to full-length PNPLA7-EGFP in our immunoblots (Fig. 3*D*). This was accompanied by the generation of a ∼25-kDa fragment likely representing EGFP, which is known to be resistant to proteinase K digestion, indicating proteolytic degradation and therefore cytosolic orientation of the C terminus of PNPLA7 (Fig. 3*D*). Using antibodies directed toward the C domain or the R domain of PNPLA7, we failed to detect additional protein bands eluding proteolytic digestion of PNPLA7-EGFP, suggesting that these domains are also largely exposed to the cytosol and thus accessible to proteolytic degradation (Fig. 3*D*). Integrity of the isolated microsomes was confirmed by the apparent protection of the 66-kDa luminal domain of Calnexin from proteolytic degradation (28) (Fig. 3*D*). To address whether the N terminus of PNPLA7 is oriented toward the cytosol or the lumen, we repeated the proteinase protection assay with microsomes isolated from COS-7 cells expressing PNPLA7 harboring an N-terminal HA tag (HA-PNPLA7). As shown in Fig. 3*E*, proteinase K treatment depleted the signal corresponding to full-length HA-PNPLA7 and led to the accumulation of an immunoreactive peptide of less than 10 kDa consistent with a luminal orientation of the N terminus. Addition of Triton X-100 to solubilize microsomal membranes rendered this peptide susceptible to proteolytic digestion, suggesting that microsomal integrity is indeed required to protect the N-terminal part of PNPLA7 from proteolysis. In an independent approach, we permeabilized COS-7 cells expressing HA-PNPLA7 either with Triton X-100 or digitonin and costained the cells with antibodies directed toward the HA tag and the C domain (Fig. 3*F*). Permeabilization with Triton X-100 rendered both epitopes accessible to antibody staining, resulting in a reticular staining pattern reminiscent of the ER and an apparent overlap of both signals. In contrast, digitonin treatment, which is used to selectively permeabilize the plasma membrane, rendered only the C region but not the N-terminal HA tag accessible to antibody staining (Fig. 3*F*). Taken together these results suggest that PNPLA7 is a bilayer-spanning TM protein that orients its N terminus toward the lumen but exposes its R and C domains largely toward the cytosol.

### The N-terminal region targets PNPLA7 to the ER

**Figure 4.**
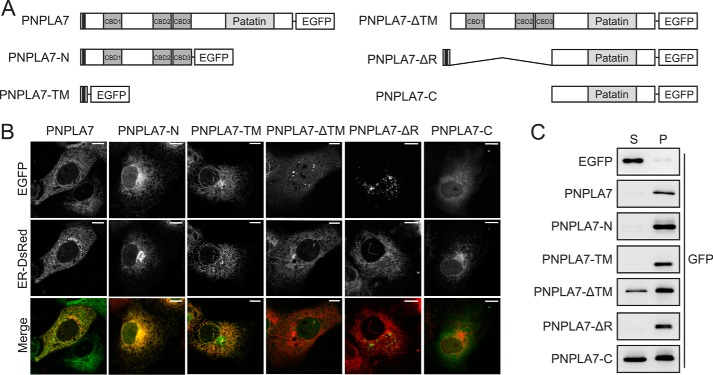
**Functional contribution of PNPLA7 protein domains to ER targeting.** *A*, domain architecture of PNPLA7 variants used in this experiment. *B*, subcellular distribution of PNPLA7 variants. COS-7 cells were cotransfected with PNPLA7-EGFP or PNPLA7-EGFP truncation mutants and a recombinant DsRed2-tagged marker of the ER and analyzed by confocal fluorescence microscopy. *Scale bars*, 10 μm. *C*, subcellular distribution of PNPLA7 variants in soluble and membrane fractions. COS-7 cells were transfected with PNPLA7-EGFP or PNPLA7-EGFP truncation mutants, separated into cytosol and membranes by ultracentrifugation, and analyzed by immunoblotting using an antibody against GFP. *S*, soluble; *P*, pellet.

We next addressed the functional contribution of individual protein domains to the subcellular positioning of PNPLA7. To this end, we constructed EGFP-tagged PNPLA7 variants lacking individual domains (Fig. 4*A*) and assessed their subcellular localization in living COS-7 cells by confocal fluorescence microscopy. Consistent with the results described above, ectopically expressed full-length PNPLA7-EGFP exhibited a reticulate pattern that colocalized with an ER marker protein in COS-7 cells (Fig. 4*B*). A PNPLA7 mutant lacking the C domain (PNPLA7-N-EGFP) exhibited a similar subcellular distribution as full-length PNPLA7-EGFP and colocalized with the ER, indicating that the C domain is dispensable for ER association of PNPLA7. Furthermore, sole expression of the N-terminal TM domain (PNPLA7-TM-EGFP) was sufficient to target EGFP to the ER, whereas deletion of the TM domain (PNPLA7-ΔTM-EGFP) compromised ER localization of PNPLA7, suggesting an essential requirement of the TM domain for ER targeting of PNPLA7. Notably, expression of a construct harboring an internal deletion of the R but not the TM domain resulted in aggregation of the recombinant protein that failed to properly distribute to the ER network. This suggests that both the TM domain and the R domain contribute to the positioning of full-length PNPLA7 at the ER. Consistent with these findings, a mutant lacking both the TM and R domains (PNPLA7-C-EGFP) distributed diffusely in the cytoplasm and showed only minimal overlap with the ER marker (Fig. 4*B*). As a complementary approach, we used subcellular fractionation and immunoblotting to assess membrane association of PNPLA7 mutant proteins. As shown in Fig. 4*C*, PNPLA7-EGFP, PNPLA7-N-EGFP, PNPLA7-TM-EGFP, and PNPLA7-ΔR-EGFP were recovered exclusively in the membrane fraction, whereas PNPLA7-ΔTM-EGFP and PNPLA7-C-EGFP distributed between the soluble and particulate fractions, suggesting impaired membrane association of these mutants. Thus, our data suggest that the N-terminal TM domain mediates efficient membrane tethering of PNPLA7 and that both the TM and R domains but not the C domain control the subcellular positioning of PNPLA7 at the ER.

### The C domain of PNPLA7 associates with LDs

**Figure 5.**
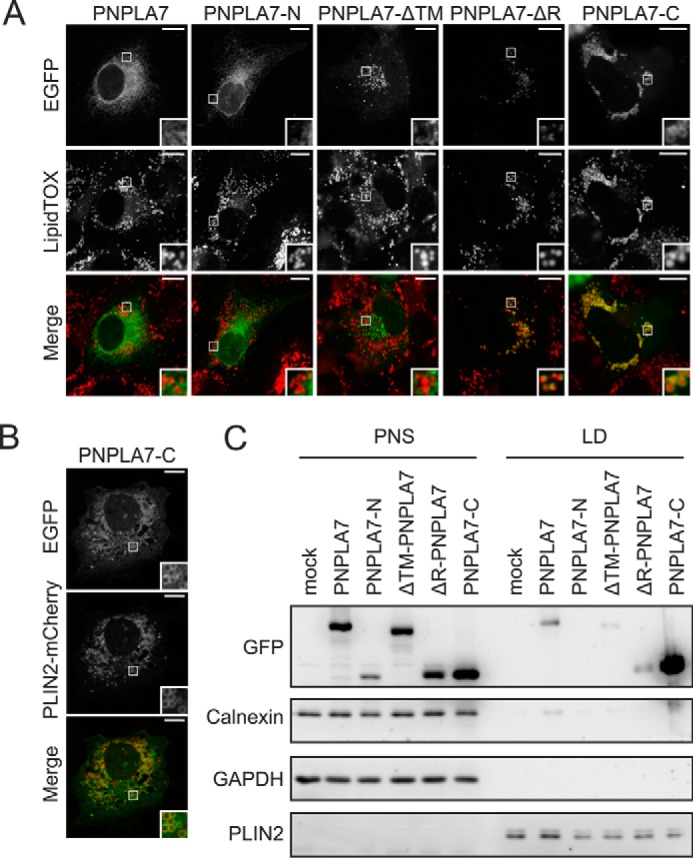
**Functional contribution of PNPLA7 protein domains to LD interactions.** *A* and *B*, subcellular distribution of PNPLA7 variants and LDs. COS-7 cells were transfected with PNPLA7-EGFP or truncated PNPLA7-EGFP mutants, incubated with FAs to induce LD formation, and analyzed by confocal fluorescence microscopy. LDs were visualized using the neutral lipid stain HSC LipidTOX Deep Red (*A*) or by cotransfection with PLIN2-mCherry (*B*). *Scale bars*, 10 μm. *C*, cofractionation of PNPLA7-EGFP or truncated PNPLA7-EGFP variants with isolated LDs. COS-7 cells were transfected with PNPLA7-EGFP or truncated PNPLA7-EGFP mutants and incubated with FAs to induce LD formation. LDs were isolated by ultracentrifugation, and the abundance of proteins in cellular fractions was assessed by immunoblotting using antibodies against GFP, PLIN2, GAPDH, and Calnexin.

In addition to the ER, PNPLA7 has been shown to localize to a subset of cellular LDs upon incubation of cells with FAs (25). To address the structural requirements for LD association of PNPLA7, we induced LD formation in COS-7 cells transfected with EGFP-tagged PNPLA7 or PNPLA7 truncation mutants and reassessed subcellular localization of the recombinant proteins by confocal fluorescence microscopy. PNPLA7-EGFP exhibited a reticular distribution and only marginal overlap with LDs, suggesting that PNPLA7 remains predominantly ER-associated also upon increased LD formation (Fig. 5*A*). Likewise, neither PNPLA7-N-EGFP nor PNPLA7-ΔTM-EGFP exhibited apparent colocalization with LDs under these conditions. In contrast, PNPLA7-ΔR-EGFP showed punctate and semi-ringlike enrichments in close proximity to LDs, indicating a possible interaction of this truncation mutant with LDs. This phenomenon was even more pronounced for PNPLA7-C-EGFP, which presented as numerous ring-shaped structures surrounding cellular LDs (Fig. 5*A*). Moreover, PNPLA7-C-EGFP colocalized with ectopically expressed PLIN2-mCherry, a *bona fide* LD protein, suggesting that it indeed associates with the LD surface (30, 31) (Fig. 5*B*).

To further study the interactions of PNPLA7 protein domains with LDs, we fractionated transfected COS-7 cells by ultracentrifugation and assessed the abundance of each PNPLA7 variant in the LD fraction by immunoblotting. Consistent with the microscopy data, ectopically expressed PNPLA7-C-EGFP was markedly enriched in the LD fraction, resembling the distribution of endogenous PLIN2 (Fig. 5*C*). Low levels of PNPLA7-EGFP, PNPLA7-ΔTM-EGFP, and PNPLA7-ΔR-EGFP were also detected in the LD fraction. However, the abundance of these proteins was much lower as compared with PNPLA7-C-EGFP.

**Figure 6.**
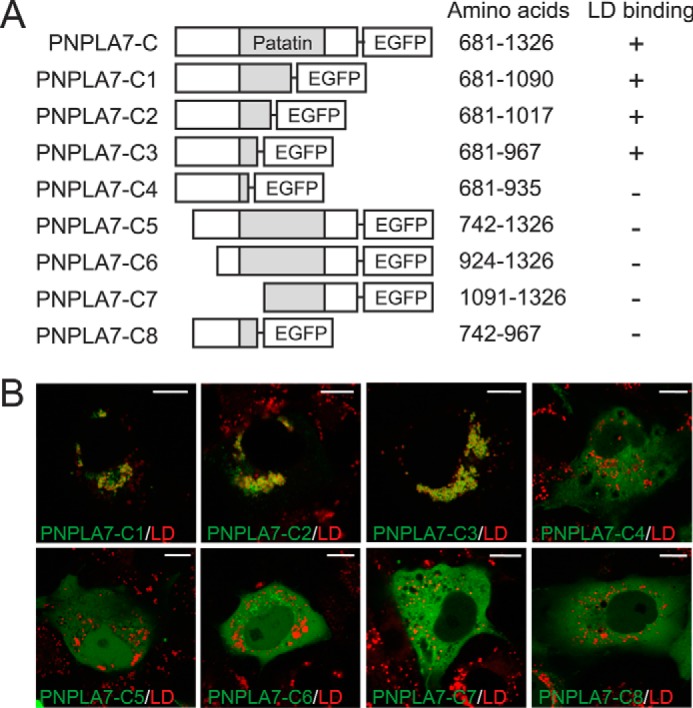
**Determination of the minimal domain required for association of PNPLA7-C with LDs.** *A*, schematic overview of PNPLA7-C-EGFP and truncation mutants and their association with LDs. *B*, subcellular localization of PNPLA7 truncation mutants and LDs. PNPLA7-EGFP truncation mutants were expressed in COS-7 cells, formation of LDs was induced by incubation with FA, and the subcellular localization of the recombinant proteins was assessed by confocal fluorescence microscopy. LDs were visualized using HSC LipidTOX Deep Red. *Scale bars*, 10 μm.

To identify the minimal domain required for LD targeting of PNPLA7-C-EGFP, we further truncated the protein and assessed the ability of these mutants to colocalize with LDs. Schematic drawings of the truncations and their effects on LD targeting are summarized in Fig. 6*A*. Mutants containing truncations at the C terminus retained the ability to localize to LDs, suggesting that the patatin-like phospholipase domain is largely dispensable for LD targeting. In contrast, even minor truncations at the N terminus of the C domain compromised LD targeting. As shown in Fig. 6, *A* and *B*, a stretch between amino acids 681 and 967 was sufficient for LD targeting, whereas further N- or C-terminal truncations abolished LD association of the C domain. Thus, a stretch of 287 amino acids is essential for the interactions between the C domain of PNPLA7 and LDs.

**Figure 7.**
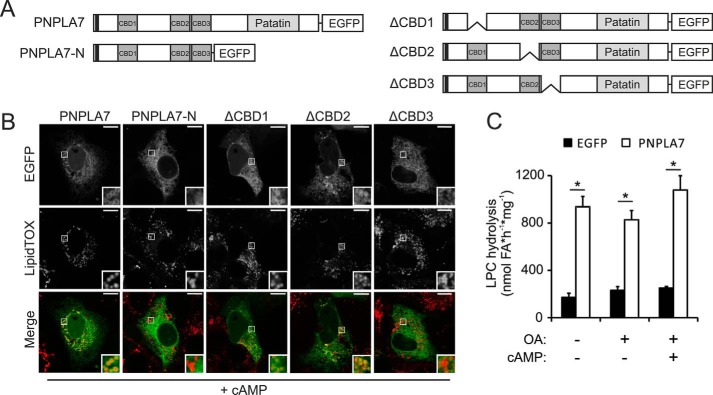
**Effect of cAMP analogs on the subcellular distribution and enzymatic activity of PNPLA7.** *A*, domain architecture of PNPLA7 variants used in this experiment. *B*, subcellular distribution of PNPLA7 variants in the presence of cAMP analogs. COS-7 cells were transfected with PNPLA7-EGFP or PNPLA7-EGFP truncation mutants, incubated with 8-CPT-cAMP and FAs, and analyzed by confocal fluorescence microscopy. LDs were visualized using HSC LipidTOX Deep Red. *Scale bars*, 10 μm. *C*, enzymatic activity of PNPLA7 in the presence of cAMP analogs. COS-7 cells were transfected with PNPLA7-EGFP or EGFP and treated with 8-CPT-cAMP and/or oleic acid (*OA*) as indicated. PNSs were incubated with C18:1 LPC as substrate, and the release of FAs was measured. Data are representative of two independent experiments and are expressed as means (*n* = 3). *Error bars* represent S.D. Statistical significance was determined using Student's unpaired *t* test. *, *p* < 0.05.

Because the N-terminal region contains three putative CBDs (Fig. 7*A*), we next asked whether the presence of cNMP analogs would affect the subcellular distribution of PNPLA7. Incubation of cells with 8-CPT-cAMP provoked a subcellular redistribution of ectopically expressed PNPLA7-EGFP, which manifested as ring- and semi-ringlike enrichments of the protein close to the LD surface on top of its reticulate ER-like distribution (Fig. 7*B*, *inset*). A similar response was elicited by 8-CPT-cGMP (data not shown). In contrast to PNPLA7-EGFP, PNPLA7-N-EGFP was not enriched at LDs under identical conditions. Furthermore, deletion of CBD3 but not CBD1 or CBD2 abolished LD interactions of PNPLA7-EGFP (Fig. 7, *A* and *B*). In summary, these data show that the C domain localizes to cellular LDs and suggest an involvement of both the C domain and CBD3 in LD interactions of PNPLA7 in response to elevated cNMP levels. Noteworthy, the specific C18:1 LPC hydrolase activity of PNPLA7 was largely unchanged upon incubation of cells with FAs and/or cNMPs (Fig. 7*C*) or starvation (data not shown), suggesting that alterations in subcellular distribution do not alter PNPLA7 activity.

### The C domain mediates catalytic activity of PNPLA7

**Figure 8.**
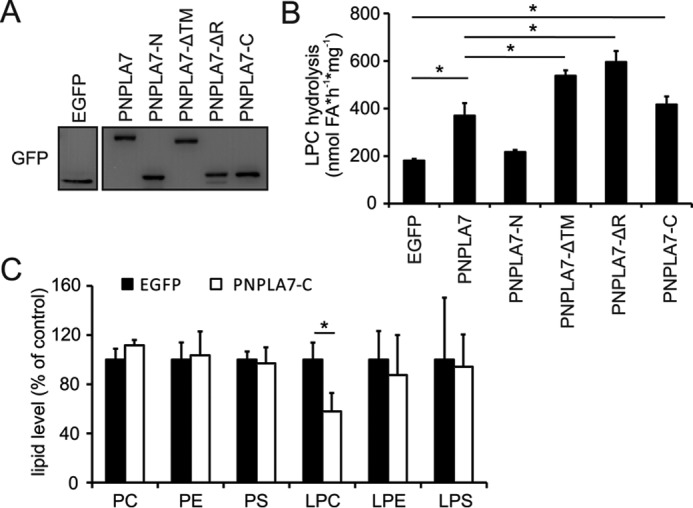
**Functional contribution of protein domains to the catalytic activity of PNPLA7.** *A*, prv tein levels of PNPLA7 variants in COS-7 cells. Cells were transfected with PNPLA7-EGFP or truncated mutants, and protein levels were analyzed in PNSs by immunoblotting using an antibody against GFP. *B*, LPC hydrolase activities of PNPLA7 and truncated mutants. PNSs of COS-7 cells transfected with PNPLA7-EGFP or truncated variants were incubated with C18:1 LPC as substrate, and the release of FA was determined. *C*, lipid levels of COS-7 cells stably expressing PNPLA7-C-EGFP or EGFP. Total lipids were extracted, and the cellular levels of the indicated lipids were analyzed by LC/MS. Data are representative of two independent experiments and are expressed as means (*n* = 3). *Error bars* represent S.D. Statistical significance was determined using Student's unpaired *t* test. *, *p* < 0.05.

We finally aimed to understand how specific protein domains regulate the enzymatic function of PNPLA7. To do so, we expressed PNPLA7-EGFP or truncated PNPLA7-EGFP mutant proteins in COS-7 cells and compared cellular lysophospholipase activities. Immunoblotting revealed similar expression levels of PNPLA7-EGFP and truncated versions of the protein (Fig. 8*A*). As shown in Fig. 8*B*, ectopic expression of PNPLA7-EGFP or PNPLA7-C-EGFP increased cellular LPC hydrolase activity to a similar extent (2.1–2.3-fold) as compared with EGFP-expressing controls, suggesting that the C domain maintains full catalytic competence also in the absence of TM and R domains. The specific activities of PNPLA7-ΔTM-EGFP and PNPLA7-ΔR-EGFP were 3.0- and 3.3-fold higher than the activity of EGFP-expressing controls, thereby moderately exceeding the activity of PNPLA7-EGFP. In contrast, expression of PNPLA7-N-EGFP failed to increase cellular lysophospholipase activity, which is consistent with a requirement of the C domain for substrate hydrolysis (Fig. 8*B*). To assess whether the catalytic C domain is functional also in intact cells, we generated COS-7 lines stably expressing PNPLA7-C-EGFP or EGFP, respectively, and performed lipid analyses. Expression of PNPLA7-C-EGFP reduced cellular levels of LPC by 42% compared with EGFP-expressing control cells but did not affect cellular levels of PC, PE, PS, LPE, and LPS (Fig. 8*C*). Thus, the C domain is both required and sufficient to mediate catalytic activity of PNPLA7 in mammalian cells.

## Discussion

In this study, we assessed the role of PNPLA7 in cellular lipid metabolism and analyzed the structure–function relationships between domain architecture, subcellular distribution, and enzymatic activity of the protein. Ectopic expression of PNPLA7 markedly increased cellular hydrolase activities toward lysophospholipids including LPC, LPE, and LPS. In contrast, phospholipids with two acyl chains were poor substrates for PNPLA7, suggesting a preference of the enzyme for mono- over diacylglycerophospholipids. In line with this observation, stable expression of PNPLA7 in COS-7 cells decreased cellular levels of the monoacylglycerophospholipid LPC but not of diacylglycerophospholipids such as PC, PE, and PS. Despite increased LPE and LPS hydrolase activities, the cellular levels of these lysophospholipid classes were also not altered upon PNPLA7 expression, arguing for a preference of PNPLA7 for LPC in a cellular context. Moreover, stable reduction of PNPLA7 expression by RNAi decreased cellular LPC hydrolase activity in AML12 cells, further supporting an enzymatic function of PNPLA7 as LPC hydrolase. PNPLA7 is closely related to PNPLA6, a protein involved in axon maintenance and brain function, which has been previously shown to regulate hydrolysis of cellular LPC and PC (11, 14, 22). Our data classify PNPLA7 also as LPC hydrolase, suggesting a function in PC/LPC turnover similar to that of PNPLA6. PC is the most abundant glycerophospholipid in mammalian membranes and serves a plethora of functions including maintenance of membrane structure, vesicle transport, and lipid signaling (32–34). Cellular PC levels are tightly regulated by synthetic and degradative processes (34). In addition, PC species composition is continuously adjusted by a remodeling process termed Lands' cycle that involves deacylation of PC to LPC and subsequent reacylation of LPC by acyltransferases (35). PNPLA6 and PNPLA7 may thus affect both bulk PC/LPC degradation and PC remodeling in the Lands' cycle by controlling the availability of LPC precursors. The striking similarities in the enzymatic activities of PNPLA6 and PNPLA7 raise the question as to what extent both enzymes fulfill distinct or redundant roles in a physiological context. Stable knockdown of PNPLA7 expression did not provoke an accumulation of cellular LPC in AML12 cells. This is likely due to the presence of additional LPC hydrolases that contribute substantial “PNPLA7-independent” LPC hydrolase activity. Notably, both PNPLA6 and PNPLA7 are expressed in AML12 cells, raising the possibility that these enzymes act in redundancy to control LPC catabolism in this particular cell line. Preliminary data from our laboratory suggest differential expression of PNPLA6 and PNPLA7 in various murine cell lines and tissues, suggesting that the contribution of each enzyme to cellular LPC hydrolysis depends on the specific cellular context. Moreover, the neurodegenerative phenotypes provoked by PNPLA6 deficiency in humans and mice that cannot be compensated by PNPLA7 argue against a redundant function of both proteins *in vivo* (14, 22, 36). Noteworthy, PNPLA7 is expressed at high levels in several non-neuronal tissues such as muscle, adipose tissue, and testis (25). Thus, PNPLA6 and PNPLA7 may perform similar biochemical functions but act in different cell types or tissues.

We initially described PNPLA7 as a lipid hydrolase with a dual subcellular localization at the ER and LDs (25). Our new data show that PNPLA7 interacts with both organelles through distinct domains. ER targeting of PNPLA7 relies on a single bilayer-spanning TM domain close to the N terminus, which is both required and sufficient for ER membrane association. In the context of the full-length protein, also the R domain contributes to the ER positioning of PNPLA7 as its deletion results in protein aggregation and perturbed distribution within the ER network. The interaction of PNPLA7 with LD does not require N-terminal ER targeting information but is instead mediated by a 287-amino acid region within its C domain. Sole expression of this region conferred LD targeting information to EGFP, whereas loss of the C domain precluded LD interactions of PNPLA7. Notably, the isolated C domain exhibited higher affinity to LDs than full-length PNPLA7. Upon increased FA supply, the C domain translocated to the LD surface, whereas full-length PNPLA7 largely retained its ER-bound configuration. Unlike the C domain, full-length PNPLA7 only partially redistributed toward LDs under specific conditions, *e.g.* in response to cNMP analogs. It is reasonable to assume that the bilayer-spanning topology of full-length PNPLA7 firmly anchors the protein within the ER and prevents translocation of the C domain to the LD surface. The domain architecture of full-length PNPLA7 may thus restrict LD interactions to specific stimuli (*e.g.* cNMP) and/or a specific subset of LDs (*e.g.* ER-associated or “nascent” LDs).

Additional structure–function analyses revealed that the C domain constitutes the major catalytic part of PNPLA7 and thus serves a dual role in subcellular localization and substrate hydrolysis. Expression of full-length PNPLA7 or the isolated C domain increased cellular lysophospholipase activity to a similar extent, suggesting that N-terminal domains neither restrict nor promote catalytic activity of PNPLA7. Consistent with this finding, it has been shown that the C-terminal esterase domain of the homologous human protein PNPLA6 acquires catalytic competence also in the absence of N-terminal regions (37). Sole expression of the C domain was sufficient to provoke a decrease in cellular LPC levels, suggesting enzymatic activity of this domain also in living cells. The combination of LD targeting information and enzymatic activity within the C domain indicates that PNPLA7 may directly act at the LD surface to degrade excess (lyso)phospholipids or antagonize phospholipid (re)synthesis. Although the functional relevance of this reaction is currently unknown, it is possible that (lyso)phospholipid catabolism contributes to distinct aspects of LD biology such as “budding” of LDs from the ER membrane or degradation of the LD monolayer in times of increased LD catabolism. In summary, our study annotates PNPLA7 as an intracellular LPC hydrolase with specific functional domains that coordinate substrate hydrolysis, ER targeting, and interactions with LDs.

## Experimental procedures

### Materials

C18:0 LPC (1-stearyl-2-hydroxy-*sn*-glycero-3-phosphocholine), C16:0 LPC (1-palmitoyl-2-hydroxy-*sn*-glycero-3-phosphocholine), C18:1 LPC (1-oleoyl-2-hydroxy-*sn*-glycero-3-phosphocholine), C18:1 LPS (1-oleoyl-2-hydroxy-*sn*-glycero-3-phospho-l-serine), C18:1 LPE (1-oleoyl-2-hydroxy-*sn*-glycero-3-phosphoethanolamine), C18:1/C18:1 PC (1,2-dioleoyl-*sn*-glycero-3-phosphocholine), C18:1/C18:1 PE (1,2-dioleoyl-*sn*-glycero-3-phosphoethanolamine), and C18:1/C18:1 PS (1,2-dioleoyl-*sn*-glycero-3-phospho-l-serine) were obtained from Avanti Polar Lipids (Alabaster, AL). l-α-[palmitoyl-9,10-^3^H]Lysophosphatidylcholine (ART1809) was obtained from American Radiolabeled Chemicals (St. Louis, MO). HCS LipidTOX^TM^ Deep Red was obtained from Thermo Fisher Scientific (Waltham, MA). 8-CPT-cAMP was purchased from Abcam (Cambridge, UK), and 8-CPT-cGMP was purchased from Sigma-Aldrich.

### Antibodies

A rabbit anti-GFP antibody (ab6556) was purchased from Abcam. A rabbit anti-PNPLA7 antibody (HPA009130), which is directed to the C domain of PNPLA7, and a mouse anti-HA antibody (H3663) were obtained from Sigma-Aldrich. A guinea pig anti-PLIN2 antibody (GP40) was obtained from PROGEN (Frankfurt, Germany). A rabbit anti-DDHD2 antibody (25203-1-AP) was obtained from Proteintech (Manchester, UK). Rabbit anti-Calnexin (2679), rabbit anti-GAPDH (2118), and rabbit anti-IRE1α (3294) antibodies were obtained from Cell Signaling Technology (Danvers, MA). Antisera against the R domains of murine PNPLA7 and PNPLA6 were generated by repetitive immunizations of rabbits with the peptides CSVPLPSNHGEVDELRQSQGSGSNT and PAGDPVKPTSLEAPPAPLLSRC, respectively, which were coupled to keyhole limpet hemocyanin as carrier (PiChem, Graz, Austria). An ECL^TM^ sheep anti-mouse IgG antibody (NA931) linked to horseradish peroxidase (HRP) was purchased from GE Healthcare. An HRP-linked goat anti-rabbit IgG antibody (PI-1000) was purchased from Vector Laboratories (Burlingame, CA). An HRP-linked goat anti-guinea pig IgG antibody (6090-04) was purchased from Southern Biotechnology (Birmingham, AL). A Rhodamine Red^TM^-X-linked goat anti-mouse IgG antibody was obtained from Jackson ImmunoResearch Laboratories (West Grove, PA), and DyLight® 488-conjugated goat anti-rabbit IgG antibody was obtained from Thermo Fisher Scientific.

### Protein sequence analysis

Protein domains were analyzed using the protein family database (Pfam), an online database containing collections of protein domains and families (38). TM domains were predicted using Hidden Markov Models (39).

### Plasmids and cloning of recombinant proteins

The vectors pDsRed2-ER, pmCherry-N1, and pEGFP-N3 were obtained from Clontech. pEGFP-N1 containing the coding sequence (cds) of human PLIN2 was a kind gift from Stefan Höning (Cologne, Germany). To generate a plasmid encoding PLIN2-mCherry, the cds of PLIN2 was amplified using the primers 5′-GCG AAT TCG CCA TGG CAT CCG TTG CAG TTG A-3′ and 5′-CAA CCG GTC GAT GAG TTT TAT GCT CAG ATC G-3′, and the PCR product was digested with EcoRI and AgeI and ligated into the multiple cloning site of pmCherry-N1. The cds of PNPLA7 was amplified by PCR using cDNA of murine cardiac muscle as template and the primers 5′-TTC TCG AGG CCA TGG AGG AGC AGT CCC AGT CC-3′ (F1) and 5′-GCG AAT TCG ACG AAG GAT GTT CCA GTC TTG G-3′ (R1). PNPLA7 truncation mutants were generated by PCR using the primers 5′-TTC TCG AGG CCA TGG CAG AAC CCA CTC CTC AGT AC-3′ (F2) and R1 for ΔTM-PNPLA7 (Δ1–40), 5′-TTC TCG AGG CCA TGG TTG TAA CGC GAC TGA TTC ATC TC-3′ (F3) and R1 for PNPLA7-C (Δ1–680), 5′-GCG AAT TCG CCA TGG AGG AGC AGT CCC AGT C-3′ (F4) and 5′-CAG GAT CCC CGT CGT AAT CTT CTC ACT C-3′ (R2) for TM-PNPLA7 (Δ41–1326), F1 and 5′-GCG AAT TCG ACT GTG GGT ACC TGC GCT TGA TAG-3′ (R3) for PNPLA7-N (Δ681–1326), F3 and 5′-GCG AAT TCG ATC TTG CCA CAT CCG CTG GGA G-3′ (R4) for PNPLA7-C1 (Δ1–680, Δ1091–1326), F3 and 5′-GCG AAT TCG AGA AGA TGT TGC TGA TGC TGC-3′ (R5) for PNPLA7-C2 (Δ1–680, Δ1018–1326), F3 and 5′-GCG AAT TCG AAG CAA ACA GAG CAC CCA TGA A-3′ (R6) for PNPLA7-C3 (Δ1–680, Δ968–1326), F3 and 5′-GCG AAT TCG AGG CAC ACC CTC TAG CTC CAC C-3′ (R7) for PNPLA7-C4 (Δ1–680, Δ936–1326), 5′-TTC TCG AGG CCA TGT TTG CTC TGG AGC TCC AAC A-3′ (F4) and R1 for PNPLA7-C5 (Δ1–741), 5′-TTC TCG AGG CCA TGT TGG TGC TTG GAG GGG GTG GA-3′ (F5) and R1 for PNPLA7-C6 (Δ1–923), 5′-TTC TCG AGG CCA TGT CCA TGG GGG CAA AGG TTG TG-3′ (F6) and R1 for PNPLA7-C7 (Δ1–1090), and F4 and R6 for PNPLA7-C8 (Δ1–741, Δ968–1326). The PCR products were purified by agarose gel electrophoresis and inserted into the multiple cloning site of pEGFP-N3 using the XhoI and EcoRI restriction sites to create C-terminal in-frame fusions with EGFP. Internal deletion mutants of PNPLA7 were generated using the Q5® site-directed mutagenesis kit with PNPLA7-EGFP as template and the primers 5′-CCG TCG TAA TCT TCT CAC-3′ and 5′-GTT GTA ACG CGA CTG ATT C-3′ for ΔR-PNPLA7 (Δ41–680), 5′-CAG ATC ATC ATG GTC CGG-3′ and 5′-CCG AAC ATT TTT CAA CAT GTA C-3′ for ΔCBD1 (Δ144–265), 5′-GTC CTG GGC GTG GCA CAC-3′ and 5′-GTC CTT TGT GGC AGC TCT GAA G-3′ for ΔCBD2 (Δ455–565), and 5′-GTT GTA ACG CGA CTG ATT C-3′ and 5′-CAT CCT CTT CAC AAC TGT G-3′ for ΔCBD3 (Δ578–680). A DNA fragment encoding for PNPLA7 harboring an N-terminal HA tag was created by PCR using the primers 5′-TAA TCT CGA GGC CAC CAT GTA CCC ATA CGA TGT TCC AGA TTA CGC TAT GGA GGA GCA GTC CCA GTC-3′ and 5′-GAC GAA TTC GTC AGG AAG GAT GTT CCA GTC-3′. The PCR product was purified as described above and inserted into the multiple cloning site of pEGFP-N3 using the XhoI and EcoRI restriction sites. To generate lentiviral expression vectors, pEGFP-N3 constructs encoding the open reading frames for EGFP, PNPLA7-EGFP, and PNPLA7-C-EGFP were digested with XhoI and NotI, and the resulting fragments were inserted into the multiple cloning site of pLVX IRES Puro (Clontech). Mission® lentiviral pLKO.1 vectors encoding for scrambled shRNA or shRNAs targeting murine PNPLA7 were obtained from Sigma-Aldrich. Constructs used for RNAi experiments targeted the sequences CCAAGAGGATTCTGCGCTTTA (shRNA1) and CATGTCCTTGTCAGGCTATAT (shRNA2) of the PNPLA7 cds.

### Cell culture and transfection

Neuro-2a cells (ATCC CCL-131) were maintained in minimum essential medium supplemented with 2 mm l-glutamine, 1 mm sodium pyruvate, non-essential amino acids, 10% fetal bovine serum (FBS), 100 units/ml penicillin, and 100 μg/ml streptomycin. COS-7 cells (ATCC CRL-1651) were cultured in Dulbecco's modified Eagle's medium (DMEM) supplemented with 10% FBS, 100 units/ml penicillin, and 100 μg/ml streptomycin. AML12 cells (ATCC CRL-2254) were cultured in a 1:1 mixture of DMEM and Ham's F-12 medium supplemented with 10% FBS, 100 units/ml penicillin, 100 μg/ml streptomycin, 0.005 mg/ml insulin, 0.005 mg/ml transferrin, 5 ng/ml selenium, and 40 ng/ml dexamethasone. All cells were maintained at 37 °C, 95% humidity, and 5% CO_2_. Cells were transfected with Metafectene® (Biontex GmbH, Munich, Germany) according to the manufacturer's instructions and used for experiments 24 h thereafter. Cells were treated with 400 μm oleic acid bound to bovine serum albumin (BSA; essentially fatty acid-free, Sigma-Aldrich), 1 mm 8-CPT-cAMP, or 1 mm 8-CPT-cGMP for 16 h or with Hanks' buffered salt solution for 4 h before being processed for imaging or lipid hydrolase assays.

### Generation of lentivirus and stable infection of COS-7 and AML12 cells

Lentiviral particles harboring pLVX IRES Puro vectors for the expression of EGFP-tagged proteins or pLKO.1 vectors for the expression of shRNAs were generated in HEK293T cells (ATCC CRL-3216) according to the manufacturer's instructions (Clontech). Before transduction, COS-7 or AML12 cells were seeded into 6-well plates at a density of 300,000 cells/well. Cells were incubated for 24 h with lentivirus-containing supernatants in the presence of 8 μg/ml Polybrene. To select for stable expression, cells were maintained for 7 days in medium containing 2 μg/ml puromycin.

### Lipid analysis

Total lipids of cell pellets (1 × 10^6^ cells) were extracted twice according to Folch *et al.* (40) using chloroform/methanol/water (2:1:0.6, v/v/v) containing 500 pmol of butylated hydroxytoluene, 1% acetic acid, and 100 pmol of internal standards (17:0/17:0 PC, 19:0/19:0 PC, 17:0/17:0 PE, 17:0/17:0 PS, and 17:0 LPC, Avanti Polar Lipids) per sample. Extraction was performed under constant shaking for 60 min at room temperature. After centrifugation at 1,000 × *g* for 15 min, the lower organic phase was collected. 2.5 ml of chloroform was added to the remaining aqueous phase, and the second extraction was performed as described above. Combined organic phases of the double extraction were dried under a stream of nitrogen and resolved in 150 μl of methanol/2-propanol/water (6:3:1, v/v/v) for UPLC-triple quadrupole analysis. Chromatographic separation was modified after Knittelfelder *et al.* (41) using an AQUITY UPLC system (Waters) equipped with a Kinetex EVO C_18_ column (2.1 × 50 mm, 1.7 μm; Phenomenex) starting with a 25-min gradient with 100% solvent A (MeOH/H_2_O (1:1, v/v), 10 mm ammonium acetate, 0.1% formic acid). An EVOQ Elite^TM^ triple quadrupole mass spectrometer (Bruker) equipped with an electrospray ionization source was used for detection. Lipid species were analyzed by selected reaction monitoring (PC: MH^+^ to *m*/*z* 184, 25 eV; LPC: MH^+^ to *m*/*z* 184, 22 eV; PE: MH^+^ to −*m*/*z* 141, 20 eV; LPE: MH^+^ to −*m*/*z* 141, 17 eV; PS: MH^+^ to −*m*/*z* 185, 20 eV; LPS: MH^+^ to −*m*/*z* 185, 17 eV). Data acquisition was done by MS Workstation (Bruker). Data were normalized for recovery and extraction and for ionization efficacy by calculating analyte/internal standard ratios.

### Subcellular fractionation

COS-7 cells were washed with phosphate-buffered saline (PBS), scraped from tissue culture plates, and collected by brief centrifugation. For the preparation of membrane and cytosol, cells were homogenized in 10 mm HEPES, 0.25 m sucrose, 1 mm EDTA, pH 7.4 (buffer A), by passing 30 times through a 26-gauge needle on ice. PNSs were obtained by centrifugation at 4 °C and 1,000 × *g* for 10 min. PNS was further fractionated into cytosol and membrane fractions by ultracentrifugation at 4 °C and 100,000 × *g* for 60 min. For the isolation of lipid droplets, cells were homogenized on ice with a Dounce homogenizer in 20 mm Tris/HCl, 1 mm EDTA, pH 7.4, and lipid droplets were isolated by ultracentrifugation using a discontinuous sucrose gradient according to Brasaemle and Wolins (42) with minor modifications. Protein concentrations of cell extracts were determined with the Bio-Rad Protein Assay kit according to the manufacturer's instructions (Bio-Rad) using BSA as standard. Alternatively, protein concentrations were determined with the Pierce® BCA^TM^ Protein Assay kit according to the manufacturer's protocol (Thermo Scientific).

### Membrane extraction and proteinase K protection assays

Membrane fractions of COS-7 cells were resuspended in buffer A and incubated in the presence of 1 m NaCl, 0.1 m Na_2_CO_3_, or 1% Triton X-100 for 30 min on ice. Afterward, soluble and insoluble components were separated by centrifugation at 4 °C and 100,000 × *g* for 30 min and subjected to immunoblotting. For membrane protection assays, isolated membrane fractions were incubated in the presence of 0–10 μg/ml proteinase K in the absence or presence of 1% Triton X-100 for 15 min at room temperature. The reactions were stopped by the addition of PMSF at a final concentration of 2 mm, further incubated for 5 min, and subjected to immunoblotting.

### Immunoblotting

Samples were mixed with Laemmli buffer, denatured for 5 min, subjected to SDS-PAGE, and electroblotted onto PVDF membranes. Membranes were incubated for 1 h with 20 mm Tris/HCl, 150 mm NaCl, 0.1% Tween 20, pH 7.5 (TBST), containing 10% milk to block unspecific binding sites. Primary and secondary antibodies were diluted in TBST containing 5% milk and incubated with the membrane for 1 h at room temperature followed by extensive washing with TBST.

### RNA extraction and RT-qPCR

Cells were harvested and snap frozen in liquid nitrogen. RNA was extracted using the TRIzol® reagent (Invitrogen^TM^, Life Technologies) according to the manufacturer's instructions. RT-qPCR was performed as described previously (43) using the StepOnePlus^TM^ Real-Time PCR System (Applied Biosystems, Life Technologies) and the Maxima^TM^ SYBR Green/ROX PCR reaction mixture (Thermo Scientific Fermentas, Waltham, MA). The following primers were used: *Pnpla7* fw, 5′-CGT GTT TTC CAA CGA CCA CC-3′; *Pnpla7* rv, 5′-TCT GCT AGT GCC CTG AGG AT-3′; *36B4* fw, 5′-GCT TCA TTG TGG GAG CAG ACA-3′; *36B4* rv, 5′-CAT GGT GTT CTT GCC CAT CAG-3′. Relative mRNA levels were quantified according to the ΔΔCt method using *36B4* as reference gene.

### Lipid hydrolase assays

Protein samples for lipid hydrolase assays were prepared by sonication of COS-7 or AML12 cells in 0.25 m sucrose, 1 mm EDTA, 1 mm DTT containing 20 μg/ml leupeptin, 2 μg/ml antipain, and 1 μg/ml pepstatin (solution A) followed by centrifugation at 4 °C and 1,000 × *g*. Lipid substrates were prepared by sonication of glycerophospholipids in 200 mm Bistris propane buffer, pH 7, containing 10 mm CHAPS, 1 mm EDTA, and 600 mm NaCl. Unless otherwise indicated, substrates contained lysophospholipids at final concentrations of 3 mm or diacylglycerophospholipids at final concentrations of 1 mm. Assays were started by the addition of 50 μl of substrate to 50-μl samples in solution A and incubated under steady shaking at 37 °C in a water bath. Substrate hydrolysis is expressed as release of FAs, which was determined routinely using a commercially available kit (non-esterified FA (NEFA) reagent, Wako, Neuss, Germany) according to the manufacturer's instructions. For the determination of endogenous LPC hydrolase activities in AML12 cells, ^3^H-labeled LPC at a specific activity of 0.33 μCi/μmol was included in the substrate. After incubation, assays were extracted with 0.5 ml of chloroform/methanol/acetic acid (66:33:1, v/v/v), and extracts were separated by thin layer chromatography using Silica G plates and hexane/diethyl ether/acetic acid (70:30:1, v/v/v) as solvent. FAs were identified by comigrating authentic standards, and the associated radioactivity was determined by liquid scintillation counting. All assays were performed in triplicates.

### Immunostaining and confocal fluorescence microscopy

COS-7 cells were seeded in chambers mounted onto coverslips (Sarstedt, Nümbrecht, Germany) and transfected as described above. Cells expressing EGFP-tagged proteins were fixed with 4% paraformaldehyde for 20 min at room temperature, and LDs were counterstained using HCS LipidTOX Deep Red. Cells expressing HA-PNPLA7 were fixed with 4% paraformaldehyde for 20 min at room temperature and permeabilized for 15 min with PBS containing either 0.1% Triton X-100 or 0.0005% digitonin. Cells were washed three times with PBS and incubated at room temperature with PBS containing 1% BSA for 1 h to block unspecific binding. Primary antibodies were diluted in blocking solution and incubated with cells overnight at 4 °C. Cells were washed with PBS and incubated with secondary antibodies diluted in blocking solution at room temperature for 1 h followed by extensive washing with PBS. Cells were imaged using a Leica SP5 confocal microscope equipped with a Leica HCX 63× 1.4 numerical aperture oil immersion objective. EGFP and DyLight 488-conjugated antibodies were excited at 488 nm, and emission was detected between 500 and 530 nm. DsRed2, mCherry, and Rhodamine Red were excited at 561 nm, and emission was detected between 580 and 610 nm. HCS LipidTOX Deep Red was excited at 633 nm, and emission was detected between 650 and 700 nm.

### Statistical analysis

All measurements were performed in triplicates. Data are presented as means ± S.D. Statistical significance was determined by the Student's unpaired *t* test. Group differences were considered statistically significant for *p* < 0.05 (*).

## Author contributions

C. H., P.-A. C., and R. Z. conceived and coordinated the study and wrote the paper. H. X. and F. H. performed molecular biology work. C. H., B. K., and P.-A. C. performed all cellular, biochemical, and imaging experiments. T. O. E. performed lipid analyses. All authors reviewed the results and approved the final version of the manuscript.

## Footnotes

2 The abbreviations used are:

PNPLA: patatin-like phospholipase domain–containing protein
FA: fatty acid
LPC: lysophosphatidylcholine
PC: phosphatidylcholine
LPE: lysophosphatidylethanolamine
PE: phosphatidylethanolamine
LPS: lysophosphatidylserine
PS: phosphatidylserine
LD: lipid droplet
ER: endoplasmic reticulum
TM: transmembrane
OP: organophosphate
PNS: postnuclear supernatant
cNMP: cyclic nucleotide monophosphate
CBD: cNMP-binding domain
EGFP: enhanced GFP
R: regulatory
C domain: C-terminal half
8-CPT-cAMP: 8-(4-chlorophenylthio)adenosine 3′,5′-cyclic monophosphate
8-CPT-cGMP: 8-(4-chlorophenylthio)guanosine 3′,5′-cyclic monophosphate
cds: coding sequence
UPLC: ultraperformance LC
qPCR: quantitative PCR
Bis-tris propane: 1,3-bis[tris(hydroxymethyl)methylamino]propane
fw: forward
rv: reverse.
